# High ATP Production Fuels Cancer Drug Resistance and Metastasis: Implications for Mitochondrial ATP Depletion Therapy

**DOI:** 10.3389/fonc.2021.740720

**Published:** 2021-10-15

**Authors:** Marco Fiorillo, Béla Ózsvári, Federica Sotgia, Michael P. Lisanti

**Affiliations:** ^1^ Translational Medicine, School of Science, Engineering and Environment (SEE), University of Salford, Greater Manchester, United Kingdom; ^2^ The Department of Pharmacy, Health and Nutritional Sciences, The University of Calabria, Cosenza, Italy

**Keywords:** anti-oxidant capacity, ATP, bedaquiline, cancer stem cells (CSCs), dormancy, mitochondria, metastasis, multi-drug resistance

## Abstract

Recently, we presented evidence that high mitochondrial ATP production is a new therapeutic target for cancer treatment. Using ATP as a biomarker, we isolated the “metabolically fittest” cancer cells from the total cell population. Importantly, ATP-high cancer cells were phenotypically the most aggressive, with enhanced stem-like properties, showing multi-drug resistance and an increased capacity for cell migration, invasion and spontaneous metastasis. In support of these observations, ATP-high cells demonstrated the up-regulation of both mitochondrial proteins and other protein biomarkers, specifically associated with stemness and metastasis. Therefore, we propose that the “energetically fittest” cancer cells would be better able to resist the selection pressure provided by i) a hostile micro-environment and/or ii) conventional chemotherapy, allowing them to be *naturally-selected* for survival, based on their high ATP content, ultimately driving tumor recurrence and distant metastasis. In accordance with this energetic hypothesis, ATP-high MDA-MB-231 breast cancer cells showed a dramatic increase in their ability to metastasize in a pre-clinical model *in vivo*. Conversely, metastasis was largely prevented by treatment with an FDA-approved drug (Bedaquiline), which binds to and inhibits the mitochondrial ATP-synthase, leading to ATP depletion. Clinically, these new therapeutic approaches could have important implications for preventing treatment failure and avoiding cancer cell dormancy, by employing ATP-depletion therapy, to target even the fittest cancer cells.

## ATP, the Energetic Currency of Life: History, Chemistry and Biology

ATP is the vital energetic “currency” of all living things, including micro-organisms (1–11). Viruses also energetically require sufficient ATP levels, for replication in host cells.

**Figure 1:**
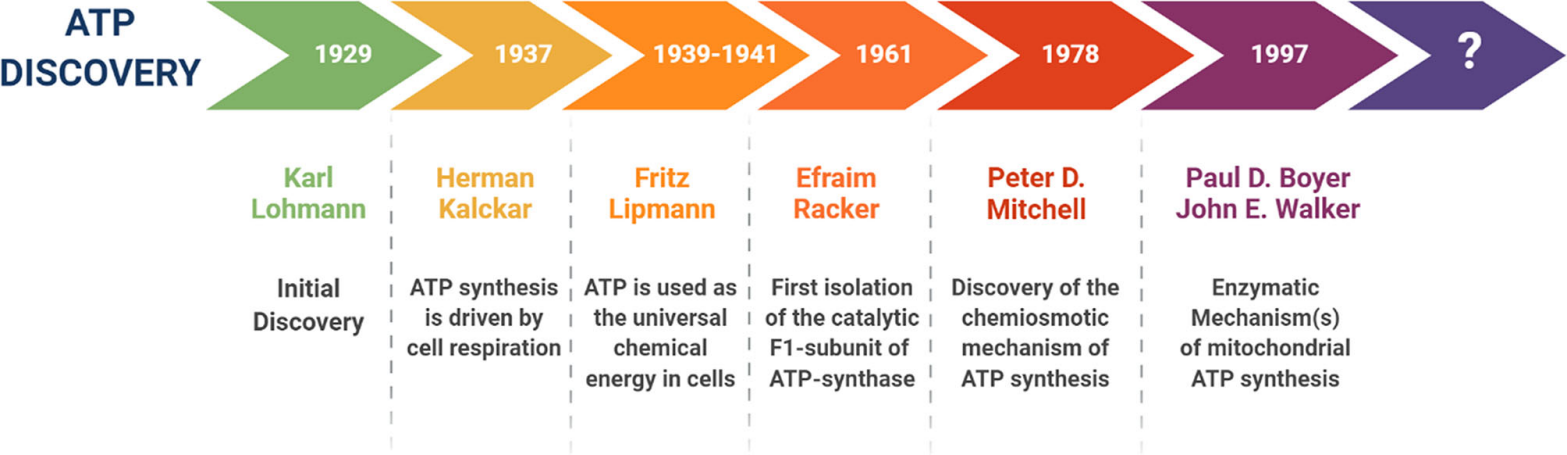
A brief history of the discovery of ATP and its energetic function. This timeline highlights the key scientists and the events that contributed to our deeper understanding the structure, function and molecular machinery responsible for the synthesis of ATP, especially within mitochondrial organelles.

Historically, ATP was initially discovered in 1929, by Karl Lohmann, a German chemist (**Figure 1**). Then, in 1937, Herman Kalckar, from Denmark, showed that ATP synthesis is driven by cell respiration. From 1939 to 1941, Fritz Lipmann, a German-born scientist, was the first to demonstrate that ATP is used as the universal chemical energy in cells. However, it wasn’t until 1961, that an American biochemist, namely Efraim Racker, first isolated the catalytic F1-subunit of the mitochondrial ATP-synthase. Then, in 1978, Peter D. Mitchell proposed that the asymmetric distribution of protons across a topologically enclosed membrane, plays an important role in mitochondrial ATP generation. In 1997, the Nobel Prize in Chemistry was jointly awarded to Paul D. Boyer and John E. Walker, for discovering the enzymatic mechanism(s), underpinning mitochondrial ATP synthesis (4–7, 12–16). The mitochondrial ATP-synthase (Complex V) is an excellent example of a rotary molecular motor, with an architecture of nanoscale dimensions (4–7).

Chemically, at the molecular level, ATP is a nucleoside triphosphate, which contains adenine, a ribose sugar, and three phosphate groups (i.e., adenosine-5’-triphosphate) (1–3). Enzymatic cleavage of ATP at its terminal phosphate group, produces two main reaction products, ADP and inorganic phosphate (Pi), thereby releasing high levels of stored chemical energy (-30.5 kJ/mole) (1–3). Importantly, free energy released by the hydrolysis of ATP is also due to the higher stability of the reaction products, because the reaction is kept away from equilibrium in living cells. As a consequence, the free energy released by the hydrolysis of ATP into ADP and Pi, is actually higher than under standard biochemical conditions.

Biologically, ATP is a required co-factor for a plethora of biochemical reactions, involved in cellular catabolism, as well as in anabolic metabolism (8–11). During passive diffusion, small molecules randomly move *via* Brownian motion, down the concentration gradient. Therefore, in living cells, in order to maintain normal physiology and organismal homeostasis, active transport is necessary to move molecules directionally and vectorially, against the concentration gradient, from an area of low concentration to an area of high concentration. This process of active transport also involves energy expenditures, in the form of ATP.

Kinases employ ATP for auto- and trans-phosphorylation reactions, to rapidly transmit information *via* cellular signaling cascades, from the plasma membrane to the cytoplasm, intracellular organelles and, ultimately, to the nucleus. The enzyme adenylate cyclase (a family of ten human genes; ADCY1-10) uses ATP as a precursor, for the generation of the second messenger, cyclic AMP (3’,5’-cyclic adenosine monophosphate).

ATP is involved in various aspects of protein synthesis. For example, tRNA-ligases employ ATP hydrolysis for coupling the 20 amino acids to their appropriate tRNAs, for their use by cellular and mitochondrial ribosomes, during protein synthesis. During protein translation, molecular chaperones (e.g., HSP70 and HSP90 family members) facilitate proper protein folding, by acting as enzymatically active ATPases, consuming large amounts of ATP.

In summary, ATP energetically “fuels” most cellular processes, including metabolism, active transport, intracellular signaling, as well as DNA, RNA and protein synthesis. Therefore, it is perhaps surprising that nutrient fasting and/or caloric restriction (17, 18) are believed to be one of the best strategies for extending both healthspan and lifespan, as evidenced by studies using model organisms (*C. elegans, Drosophila* and mice), as well as preventing cancer (19, 20). For example, Resveratrol, a natural anti-aging phytochemical and caloric restriction mimetic, is a known inhibitor of the mitochondrial ATP-synthase (21). Moreover, Resveratrol is also thought to exert its powerful anti-aging effects, *via* its sirtuin-dependent mechanisms of action.

More specifically, calorie restriction activates pro-longevity signaling pathways in model organisms, such as AMPK and the mitochondrial unfolded protein response (UPRmt), and inhibits mTOR and insulin/IGF1 signaling (22, 23). These effects may mechanistically reduce or restrict different processes that contribute to aging, such as inflammation, loss of proteostasis and senescence.

Energy for the mitochondrial synthesis of ATP is derived from the oxidation of NADH and FADH_2_ by Complexes I-IV of the mitochondrial electron transport chain (ETC). NADH and FADH_2_ are generated mainly from the TCA cycle, but some NADH is also donated by glycolysis and from the conversion of pyruvate into acetyl-CoA. However, cytosolic NADH (obtained through glycolysis) does not directly feed into the mitochondrial electron transport chain, but it gives electron equivalents *via* the malate-aspartate and/or glycerol shuttles. In contrast, NADPH, generated by the pentose-phosphate pathway (PPP), is used to maintain glutathione in a reduced state, providing anti-oxidant buffering capacity against ROS and oxidative stress.

Because of the central importance of ATP as a “barometer” of cell metabolism, many luminescent and fluorescent probes have been developed, to measure and track ATP levels, in response to various cellular stimuli (24–28). For example, BioTracker ATP-Red 1 is a vital dye that is only fluorescent when bound to ATP, but does not recognize ADP or other nutrients (29). Morphologically, BioTracker ATP-Red 1 specifically localizes to mitochondria, as seen by fluorescence microscopy, and co-localizes with the mitochondrial probe MitoTracker-Green (29). Therefore, BioTracker ATP-Red 1 allows for the dynamic detection and visualization of mitochondrial ATP in living cells and tissues.

As mitochondrial activity is specifically increased in human tumor cells and metastatic cancer cells *in vivo*, as measured by specific functional activity assays, high ATP production may be a key driving force in promoting tumor progression, therapy-resistance and, ultimately, in metastatic dissemination (30, 31). However, more mechanistic studies are needed to experimentally support this hypothesis.

## Using ATP as a Biomarker to Metabolically Fractionate the Cancer Cell Population: Implications for ATP-Depletion Therapy

In our recent studies, we took advantage of a vital fluorescent dye that allows one to measure ATP levels in living cells, namely BioTracker ATP-Red 1 (32, 33). More specifically, we coupled BioTracker ATP-Red 1 staining with a bioenergetic fractionation scheme, in which the total cell population was subjected to flow cytometry, to isolate the ATP-high and ATP-low sub-populations of MCF7 cells, an ER(+) human breast cancer cell line. This metabolic fractionation approach allowed us to isolate the most “energetic” cancer cells within the total cell population. One possibility is that increased mitochondrial metabolism and/or ROS production may contribute to this phenotype, *via* mitochondrial retrograde signalling (34, 35). Therefore, we proposed that the ATP-high cancer cell population should be targeted for eradication *via* ATP-depletion therapy (36–40). ATP-depletion therapy would be expected to result in rapid energy-depletion, especially in highly aggressive cancer cells, thereby halting their propagation, by inducing autophagy, apoptosis and/or necrosis.

**Figure 2:**
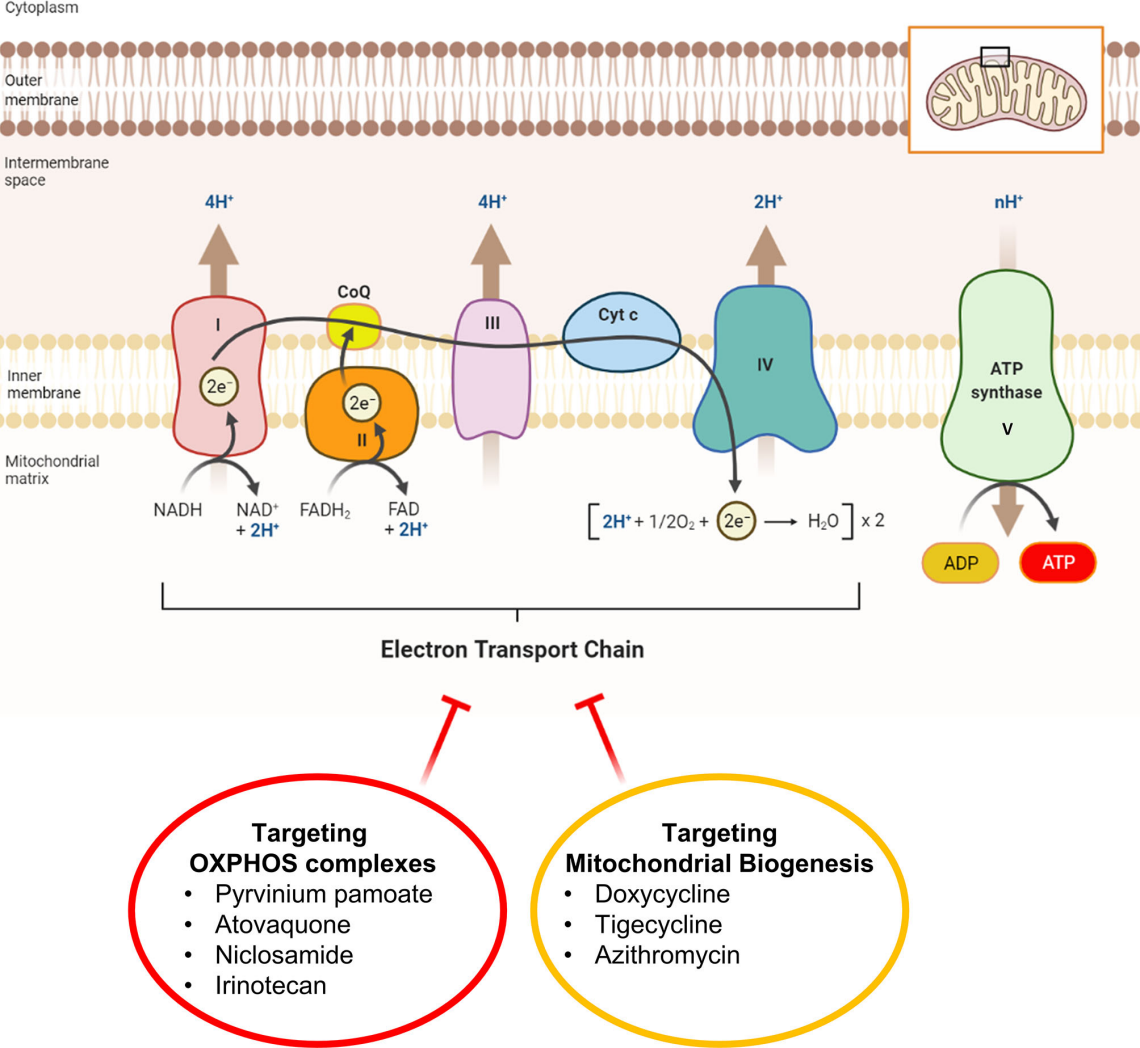
Mitochondrial complexes I to IV can be safely targeted with FDA-approved drugs. This diagram illustrates that ATP-depletion can be induced in cancer cells by employing FDA-approved mitochondrial inhibitors that either i) block OXPHOS directly or ii) block OXPHOS indirectly, by halting mitochondrial protein translation. Inhibition of mitochondrial ATP production is a manageable side-effect that can re-purposed as a therapeutic effect to target and halt the propagation of CSCs. Inhibitors of mitochondrial protein translation (Doxycycline, Tigecycline and Azithromycin) prevent the production of the 13 proteins encoded by mitochondrial DNA (mt-DNA), including key subunits of complex I, III, and IV, as well as complex V (MT-ATP6, MT-ATP8) and Humanin (MT-RNR2).

In a parallel line of research, we have previously identified >20 mitochondrially-targeted therapeutics that could be used to effectively achieve ATP-depletion therapy (**Figure 2**). These potential therapeutics include: FDA-approved drugs (Doxycycline, Tigecycline, Azithromycin, Pyrvinium pamoate, Atovaquone, Bedaquiline, Niclosamide, Irinotecan); natural products/nutraceuticals (Actinonin, CAPE, Berberine, Brutieridin, Melitidin); and experimental compounds [Oligomycin, AR-C155858, Mitoriboscins, Mitoketoscins, Mitoflavoscins, TPP derivatives (including Dodecyl-TPP and 2-Butene-1,4-bis-TPP)] (41–47). A triple-combination of two antibiotics together with Vitamin C (Doxycycline, Azithromycin and Ascorbic acid) was found to be particularly potent for targeting mitochondria, inducing ATP-depletion and inhibiting CSC propagation (48), at sub-antimicrobial levels.

As many of these are repurposed FDA-approved antibiotics, with excellent safety profiles, Phase II clinical trials are warranted. For example, a Phase II clinical pilot study of Doxycycline (49) has already shown that this >50-year-old antibiotic is indeed effective in metabolically targeting the CSC population in early breast cancer patients, as demonstrated using CD44 and ALDH1 as specific CSC markers (49). Mitochondrial ATP-depletion therapy is expected to functionally mimic fasting and/or caloric restriction, thereby more effectively starving CSCs to death. This has important implications for cancer prevention (50–52) and for potentially extending human lifespan during aging (53).

Recently, we also demonstrated that treatment with a panel of mitochondrially-targeted therapeutics, which potently inhibit mitochondrial protein translation or OXPHOS, could block tumor cell metastasis, using an *in vivo* pre-clinical model (54–56). These results indicated that ATP levels are functionally critical for the processes fueling aggressive tumor cell behaviors and spontaneous metastasis.

In further support of our hypothesis, other mitochondrial inhibitors are known to have promising anti-cancer effects, including IACS-010759, Gboxin, β1-blockers, Nebivolol, and Benzethonium (57–60).

## Survival of the “Fittest”: ATP-High Cancer Cells Show a Multi-drug Resistant Phenotype, With Enhanced Anti-oxidant Capacity

**Figure 3:**
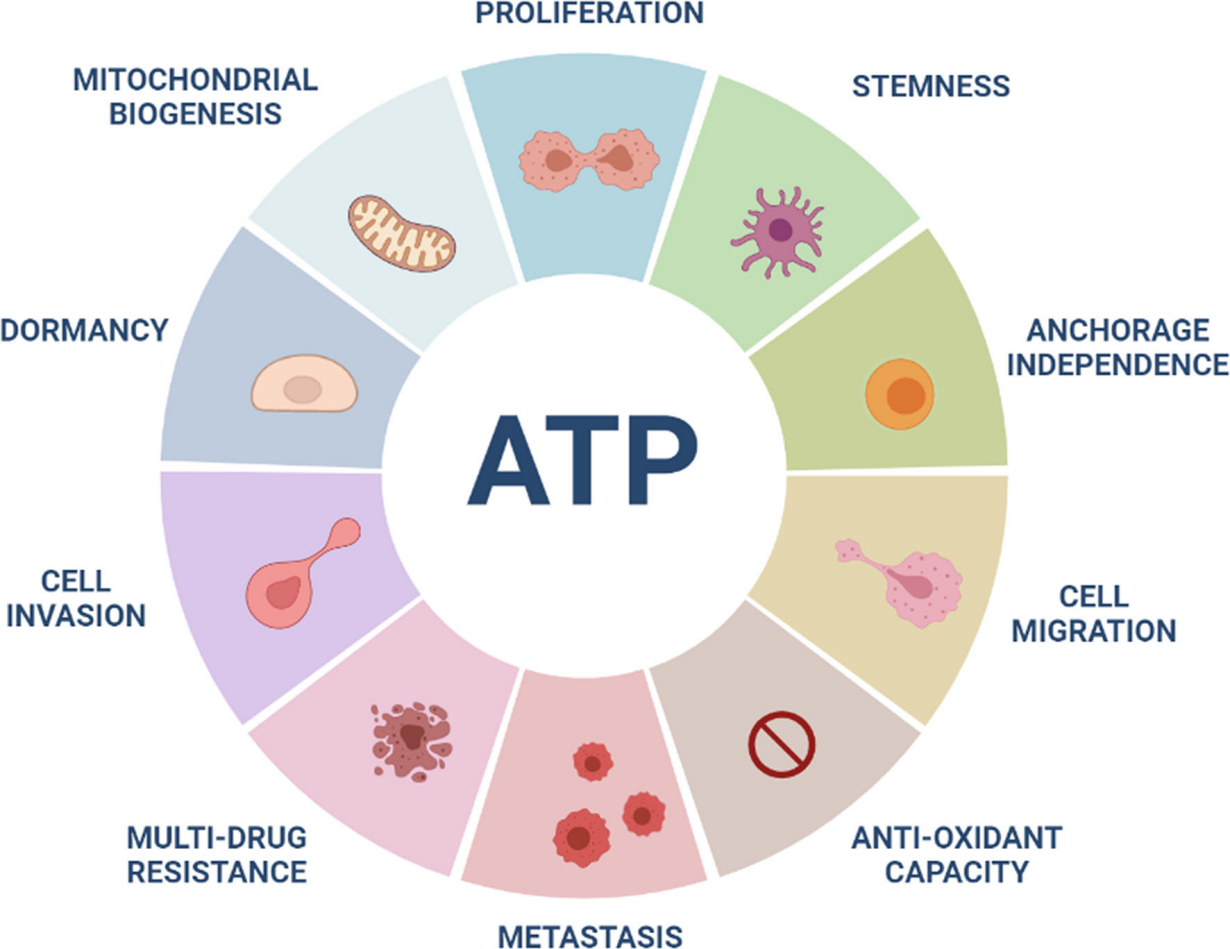
High ATP levels are a major driver of aggressive cancer cell phenotypes. ATP-high cancer cells show increases in many aggressive properties or behaviors, including cell proliferation, stemness, anchorage-independence, migration, invasion, metastasis, anti-oxidant capacity and drug-resistance. In contrast, more “dormant” CSCs show low ATP levels. High mitochondrial ATP production may be related to increases in mitochondrial mass in ATP-high cancer cells.

Previous studies have shown that high anti-oxidant capacity, due to increased levels of reduced glutathione, elevated NADPH, and activated NRF2 signaling, significantly contributes to the onset of multi-drug resistance (61–67). Consistent with this hypothesis, recently we directly showed that ATP-high MCF7 cells have an increased anti-oxidant capacity, with elevated levels of reduced glutathione, and are intrinsically resistant to four different classes of drugs (Tamoxifen, Palbociclib, Doxycycline and DPI) (33). Therefore, the existence of the ATP-high CSC phenotype may help to mechanistically explain the pathogenesis of multi-drug resistance, during cancer therapy (**Figure 3**). In this context, current cancer therapy may allow only the metabolically “fittest” cancer cells to survive.

More specifically, as we have shown that the ATP-high phenotype is indeed transient, consistent with a “stemness” phenotype, external selection pressure created by a hostile environment, such as chemo-therapy, may further stabilize this metabolic state. As such, this high energy phenotype may be required for the survival of only the “fittest” cancer cells, allowing their propagation, under these harsh conditions.

Our recent findings with ATP-high MCF7 cells are also consistent with several other studies that establish a direct causal relationship between mitochondrial “power” and Tamoxifen-resistance. For example, MCF7-TAMR cells that were generated *via* chronic exposure to increasing concentrations of Tamoxifen, resulting in Tamoxifen-resistance, showed elevated levels of mitochondrial OXPHOS and ATP production (66). In MCF7-TAMR cells, acquired Tamoxifen-resistance was due to the over-expression of two key anti-oxidant proteins (NQO1 and GCLC) and their positive metabolic effects on mitochondrial metabolism, as revealed by unbiased proteomics analysis (66). In addition, recombinant over-expression of either NQO1 or GCLC in MCF7 cells autonomously conferred an ~2-fold increase in mitochondrial ATP-production and Tamoxifen-resistance (66). Moreover, recombinant over-expression of a somatic mutation (Y537S) in the estrogen receptor (ER-alpha; ESR1), clinically associated with acquired Tamoxifen-resistance in breast cancer patients, genetically conferred elevated mitochondrial biogenesis, OXPHOS and high ATP production (68). The proteomic profiles of MCF7-TAMR cells and MCF7-ESR1(Y537S) cells also showed considerable overlap in the biological processes that were functionally activated (68). Finally, 60 gene products functionally-associated with mitochondrial ATP production, were predictive of Tamoxifen-resistance in ER(+)/Luminal A breast cancer patients (69). These predictive biomarkers included 18 different mitochondrial ribosomal proteins (MRPs) and >20 distinct components of the mitochondrial OXPHOS complexes. Therefore, our recent results showing that “naïve” ATP-high MCF7 cells are intrinsically Tamoxifen-resistant, without any prior exposure to the drug, have important clinical implications for optimizing the effectiveness of hormonal breast cancer therapy.

Interestingly, it has been previously reported that treatment with conventional chemotherapeutic regimens, actually increases the number of CSCs, while selectively killing “bulk” cancer cells (70), but no metabolic hypotheses have been proposed to explain this phenomenon. In accordance with our “ATP-based hypothesis”, Chan and colleagues (from Genentech, Inc.) examined the effects of gemcitabine and etoposide on the total cancer cell population (71). Remarkably, they observed that after treatment with gemcitabine and etoposide, the population of surviving cells showed an increase in ATP content, elevated mitochondrial mass, with more mitochondrial respiration (71). However, they did not propose a mechanistic explanation for these observations, nor did they consider the CSC population. Instead, they simply concluded that measuring ATP is not a good read-out to assess the effectiveness of chemo-therapeutic agents. Given our current findings with ATP-high cells, an alternate interpretation of their results is that gemcitabine and etoposide selectively killed the ATP-low sub-population of cancer cells, thereby enriching for the “energetic” ATP-high sub-population, which are more stem-like and drug-resistant. Therefore, new drug discovery should be initiated to help eradicate the ATP-high sub-population of cancer cells.

Higher intracellular ATP levels have also been suggested to account for acquired drug-resistance to oxaliplatin and cisplatin, in a variety of chronically-treated colon and ovarian cancer cell lines (HT29, HCT116, A2780), although a diverse number of mechanisms have been proposed, including increased glycolysis and/or mitochondrial metabolism (72, 73). However, in these previous studies, ATP levels were measured only after chronically selecting for the drug resistant cell population. Therefore, a direct cause-effect relationship between ATP production and drug resistance could not be established.

Taken together, these findings are internally consistent with the idea that the high selection pressure afforded by these conventional chemotherapeutic agents ultimately drives the natural-selection and survival of only the “energetically fittest” cancer cells, namely the ATP-high sub-population. Therefore, in the future, new drug therapies must be implemented, to target and eradicate the ATP-high population of cancer cells, to prevent the accumulation of an aggressive, metastatic sub-population of tumor cells.

## Tumor Dormancy and Multi-Drug Resistance: Are They Inter-Related?

According to the conventional view of tumor dormancy, dormant cancer cells undergo slower rates of cell proliferation and/or cell cycle arrest (quiescence), to avoid therapy-induced cell death, leading to multi-drug resistance (67, 74). Surprisingly, recently we observed just the opposite phenomenon. ATP-low MCF7 cells were less proliferative, with >87% of the cells in the G0/G1 phase of the cell cycle, but were actually more sensitive to 4 different classes of drugs, using the 3D-mammosphere assay as a readout (33). Conversely, ATP-high MCF7 cells were significantly more proliferative, with >38% of the cells in either S-phase or G2/M, showing a clear multi-drug resistance phenotype. Therefore, high levels of mitochondrial ATP appear to be a key driver of both elevated cell proliferation and drug-resistance, as they represent the energetically “fittest” population of cancer cells (**Figure 3**).

## Defining a Metastasis Gene Signature, Using Bioinformatics: Validating the Importance of ATP5F1C, Using Several Independent Data Sets and MDA-MB-231 Cells

**Figure 4:**
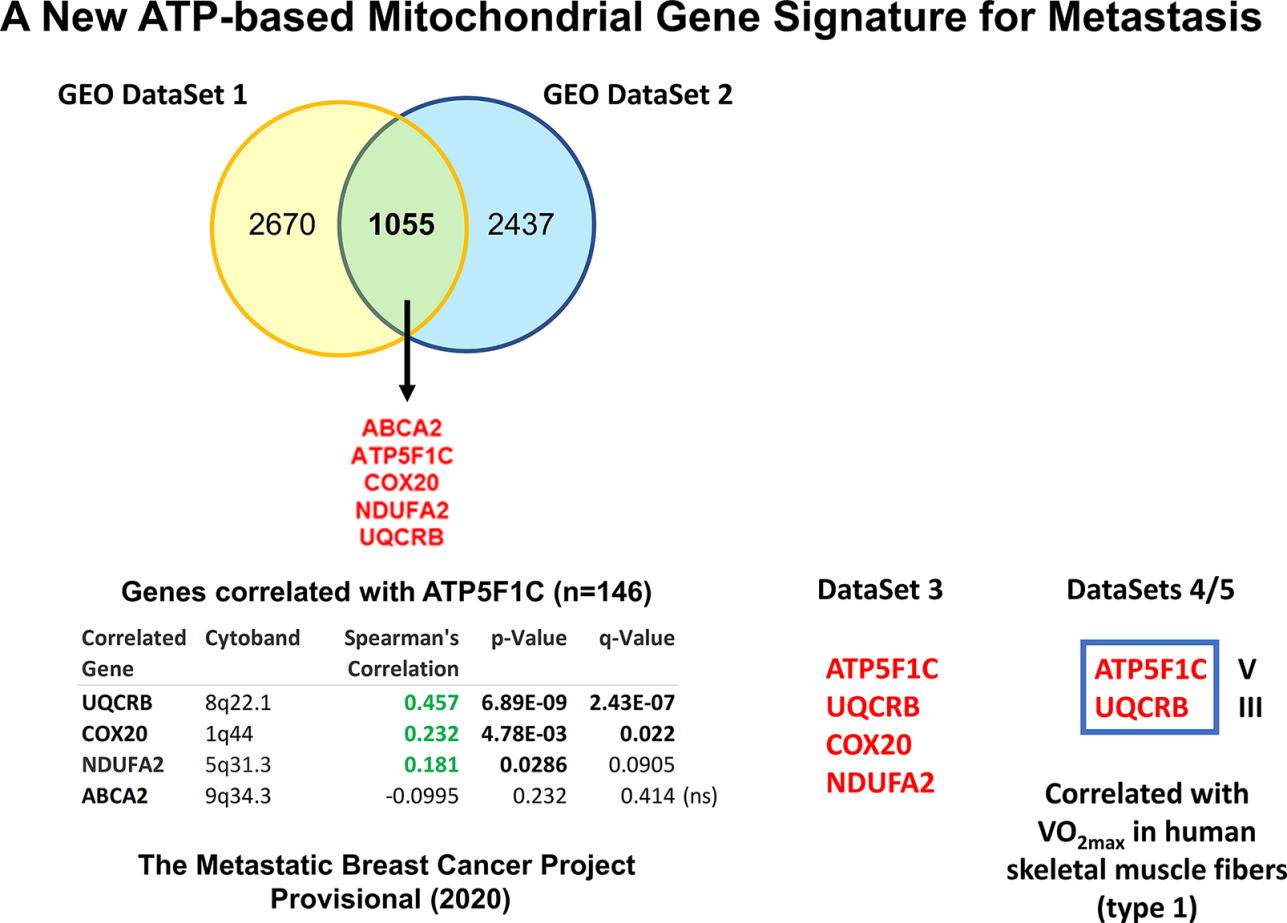
Using several independent data sets to identify ATP5F1C as a key biomarker and therapeutic target for metastasis prevention. In order to define an ATP-related metastasis gene signature we first intersected two GEO DataSets focused on breast cancer metastasis (namely, GSE2034 and GSE59000), resulting in 5 common genes. The positive co-expression of ATP5F1C, with 3 other members of this gene signature (UQCRB, COX20, NDUFA2), was indeed confirmed by analyzing data from The Metastatic Breast Cancer Project (Provisional, February 2020; DataSet 3; https://mbcproject.org). Finally, 2 of these 4 gene transcripts (ATP5F1C and UQCRB) were independently found to be specifically-associated with i) maximal oxygen uptake (VO_2max_) and ii) a higher percentage of mitochondrial-rich (type 1) fibers, in human skeletal muscle (DataSets 4/5), especially during exercise training. Therefore, ATP5F1C and UQCRB are likely to be key biomarkers of high OXPHOS and high mitochondrial ATP production in cancer cells. *Modified from Reference 33 and reproduced with permission, under a Creative Commons License*.

To interrogate the possible role of mitochondrial ATP production in the process of metastasis, we also used a bioinformatics approach (33). Briefly, we intersected a series of publicly-available GEO breast cancer DataSets and defined a metastasis-associated gene-signature consisting of five ATP-related genes, namely ATP5F1C, UQCRB, COX20, NDUFA2, and ABCA2 (**Figure 4**). Notably, two members of the signature, ATP5F1C and UQCRB, are both known markers of maximal oxygen uptake (V0_2max_) in mitochondrial-rich human skeletal muscle fibers (75).

Interestingly, ATP5F1C appeared to be the most relevant member of this metastasis signature, as it is directly connected to ATP-synthesis (76). ATP5F1C is the gamma subunit of the mitochondrial ATP synthase (Complex V) and is directly involved in converting physical energy (torque) into chemical energy (ATP) (33, 76).

To further validate and confirm the relevance of ATP5F1C, we next used a third completely independent database, namely the “The Metastatic Breast Cancer Project”, which includes mRNA expression profiling data (RNA Seq V2 RSEM) from the RNA-sequencing of metastatic breast cancer samples, derived from N=146 patients (**Figure 4**). In this context, the mRNA expression of ATP5F1C was positively correlated with the co-expression of numerous breast CSC markers, circulating tumor cell (CTC) markers, metastasis markers, cell cycle regulatory proteins, and other mitochondrial-related genes, as well as three other members of the metastasis gene signature (UQCRB, COX20, NDUFA2). Independently, using Kaplan-Meier (K-M) analysis, high levels of ATP5F1C mRNA transcripts specifically predicted poor clinical outcomes in breast, ovarian and lung cancer patients (33).

To provide functional validation, we next used MDA-MB-231 cells as a metastatic model for triple-negative breast cancer. Interestingly, ATP-high MDA-MB-231 cells over-expressed ATP5F1C, as well as other members of mitochondrial complexes I-V and CTC markers (Ep-CAM1 and VCAM1), all relative to ATP-low MDA-MB-231 cells. ATP-high MDA-MB-231 cells also showed notable increases in ATP-production, proliferation, anchorage-independent growth, cell migration, invasion and spontaneous metastasis (**Figure 3**). Conversely, inducible knock-down of ATP5F1C in MDA-MB-231 cells was indeed sufficient to inhibit ATP-production, anchorage-independent growth and cell migration.

Moreover, ATP-high sub-populations of MDA-MB-231 and MCF7 cells both showed features of multi-drug resistance, consistent with a more aggressive cancer cell phenotype.

Therefore, ATP5F1C may be an attractive target for new drug development and metastasis prevention.

## Repurposing Bedaquiline to Prevent ATP Production, Cancer Cell Motility, and Spontaneous Metastasis *In Vivo*: Targeted Down-Regulation of ATP5F1C

Are there any existing FDA-approved inhibitors of the mitochondrial ATP-synthase that could be repurposed to target and prevent cancer metastasis? This would certainly accelerate future clinical trials, as FDA-approved drugs can re-enter Phase II trials, for another clinical indication, completely skipping Phase I, which is specifically focused on safety and toxicity.

**Figure 5:**
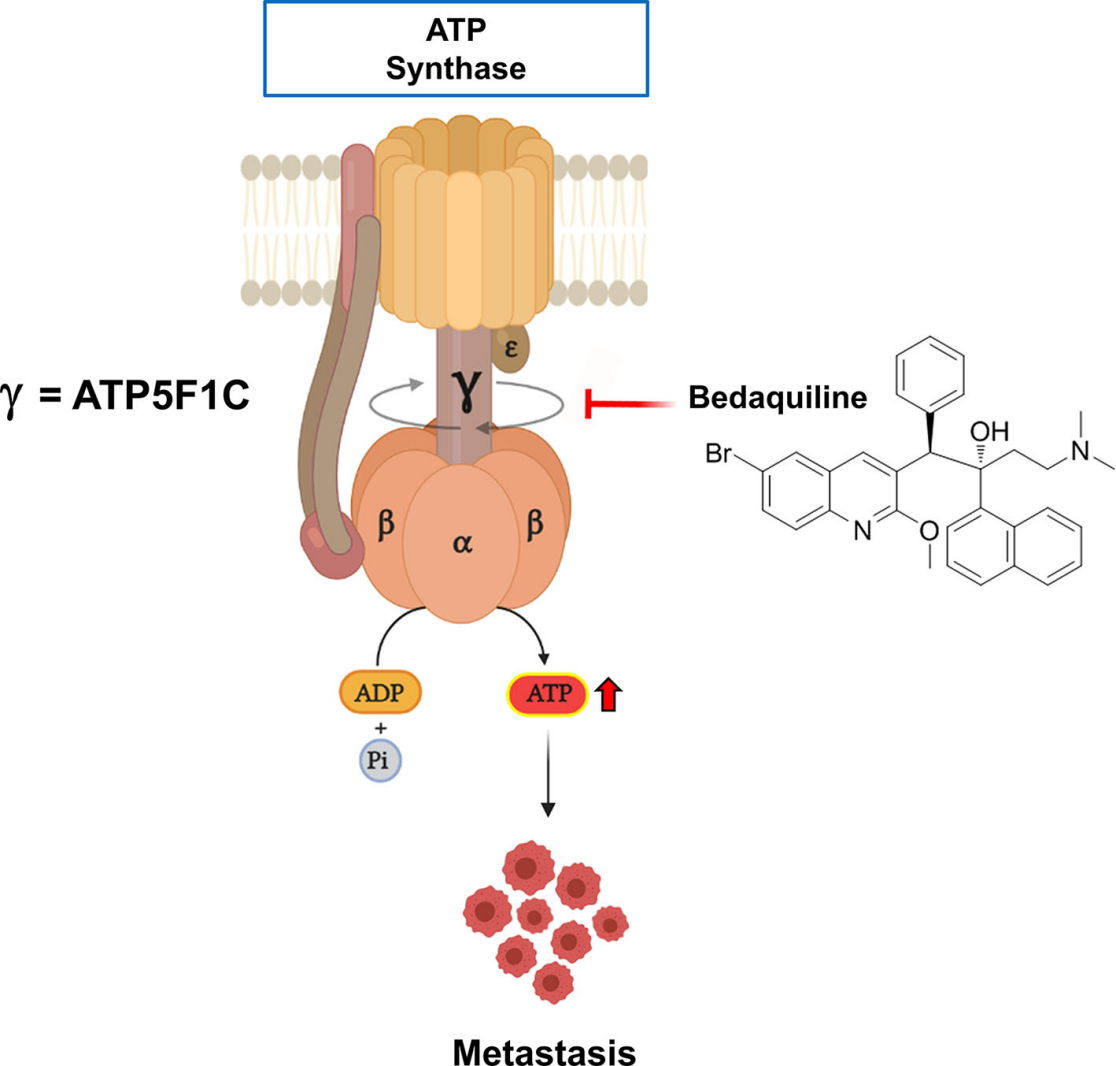
Targeting the human mitochondrial ATP synthase with Bedaquiline, an FDA-approved drug, prevents spontaneous metastasis. Mitochondrial ATP-synthase is a nano-scale rotary molecular motor that uses the transport of hydrogen ions to generate physical energy in the form of torque that is then converted into chemical energy in the form of ATP. Rotation of the gamma-subunit (ATP5F1C) helps to convert physical energy into chemical energy. Note that Bedaquiline treatment induces the degradation or down-regulation of the gamma-subunit (ATP5F1C), resulting in ATP-depletion and the prevention of metastasis.

Bedaquiline is a clinically-approved drug, that is usually used for anti-tuberculosis therapy, especially in the context of drug-resistant TB strains. More specifically, Bedaquiline was originally designed to target and block the activity of the ATP-synthase in mycobacteria. Perhaps surprisingly, recent studies have also demonstrated that Bedaquiline significantly inhibits the human and the yeast mitochondrial ATP-synthase, as an off-target side effect (77). In addition, using cryo-EM as a tool for structural studies, investigators have localized the binding site of Bedaquiline to the integral membrane subunit (F_0_), using the yeast mitochondrial ATP-synthase. Since the soluble F_1_ subunit is physically tethered to the membrane-bound F_0_ subunit *via* the gamma-subunit (ATP5F1C) (76), we hypothesized that ATP5F1C might be mis-folded and degraded in the presence of Bedaquiline. This would effectively disrupt ATP synthesis, as ATP5F1C functions as the rotating central stalk that helps convert torque into chemical energy, in the form of ATP (**Figure 5**).

Interestingly, we observed that ATP5F1C was effectively down-regulated after Bedaquiline treatment in MDA-MB-231 cells, resulting in significant reductions in ATP production, stemness, anchorage-independent growth and cell migration (33).

Bedaquiline-induced cell death in MDA-MB-231 cells was related to the onset of autophagy and necrosis, but apoptosis was not observed. Remarkably, the expression of ATP5F1C and ATP-production, as well as cell growth, remained unaffected after Bedaquiline treatment in MCF-10A cells, a non-tumor-producing human breast epithelial cell line. Therefore, the effects of Bedaquiline appeared to be restricted to cancer cells. Similarly, Bedaquiline inhibited ATP-production in MCF7 breast cancer cells, but not in hTERT-BJ1 cells, a normal human fibroblast cell line (44).

As a result of these findings, we tested the efficacy of Bedaquiline in a pre-clinical xenograft model, namely the CAM assay, which uses chicken eggs as the host for measuring tumor growth, spontaneous metastasis and drug toxicity (33). Our results demonstrated that Bedaquiline had no effect on MDA-MB-231 tumor growth, but effectively prevented spontaneous metastasis, by nearly 85%, at a concentration that did not show any significant chicken embryo toxicity (**Figure 5**).

As a consequence, we suggest that Bedaquiline could be re-purposed to prevent spontaneous metastasis, by driving ATP-depletion *via* its targeting of the ATP5F1C subunit, within the mitochondrial ATP-synthase multi-subunit complex. As such, clinical trials may be warranted.

We speculate that Bedaquiline, by mechanistically targeting the gamma-subunit of the ATP synthase, may promote dissociation of the F1-domain of the enzyme and thereby promote the opening of the transition pore (78–80). Recent findings strongly support the idea that the ATP synthase forms the permeability transition pore (PTP) (78, 79). Prolonged opening of the PTP permeabilizes the inner mitochondrial membrane to small solutes and constitutes the point of no return in the execution of cell death.

Finally, the mitochondrial ATP-synthase is indeed subjected to numerous post-translational modifications (such as phosphorylation, as well as acetylation and succinylation on key lysine residues). Of course, this can potentially affect its level of enzymatic activity, and could perhaps explain the phenotypic differences between ATP-high and ATP-low cancer cells. Regarding Bedaquiline, this FDA-approved drug is known to bind directly to the ATP-synthase, but it is not known if Bedaquiline affects the status of these post-translational modifications.

## Conclusions

In conclusion, we recently employed bioenergetic cell “stratification” using an ATP-based biomarker to isolate the metabolically “fittest” cancer cells. Using this novel approach, we obtained the first evidence that high levels of mitochondrial ATP are a primary determinant of aggressive cancer cell behavior(s), including spontaneous metastasis. These findings have important therapeutic implications for preventing treatment failure in cancer patients, which remains an urgent unmet clinical need.

For example, energetic cell profiling, using ATP as a biomarker, can provide a reliable source of ATP-high CSCs i) for establishing “living” tumor bio-banks and ii) for conducting small-molecule library screening, targeting drug resistance. This new conceptual framework will allow novel strategies to be developed to therapeutically target and eradicate even the energetically “fittest” CSCs, to ultimately abrogate drug resistance and metastasis.

In direct support of these observations, Kalluri and colleagues (81) observed that shRNA-mediated down-regulation of the key mitochondrial transcription factor, namely PGC-1α, significantly inhibited lung metastasis, in several independent cell lines (MDA-MB-231, 4T1 and B16F10 melanoma cells), but had little or no effect on tumor growth. These observations are consistent with the idea that targeted down-regulation of PGC-1α inhibited the propagation of the “fittest” CSC sub-population (31, 32, 82), although the authors did not directly address the issue of the CSC phenotype.

Similarly, high expression levels of the ATP synthase inhibitory factor 1 (IF1), which inhibits the activity of the mitochondrial ATP synthase, predicts a better outcome for breast cancer patients, especially in the case of triple-negative breast cancer (83, 84). Moreover, IF1 over-expression reduces the production of ATP in mitochondria and decreases the proliferation and invasiveness of triple-negative breast cancer cells (84).

Finally, mitochondrial DNA-encoded (mt-DNA) cytochrome c oxidase II (MT-CO2) is an essential component of mitochondrial complex IV of the respiratory chain; without MT-CO2, electron transport and mitochondrial ATP production cannot proceed. Recently, Lebok and colleagues (85) showed that high levels of MT-CO2 protein expression in a cohort of approximately 2,000 breast cancer patients, from Germany and Switzerland, were clinically associated with advanced tumor stage, higher tumor grade, lymph nodal metastasis and shorter overall survival (P < 0.0001 each). Moreover, at the molecular level, high MT-CO2 protein expression was associated with elevated Ki67 (a marker of cell proliferation), the genetic amplification of several oncogenes (HER2, MYC, CCND1 and MDM2), the deletion of PTEN (a known tumor suppressor) and the down-regulation of estrogen receptor (ER-alpha) expression (85). As MT-CO2 is a well-established surrogate marker of mitochondrial DNA content and mitochondrial protein translation, these results clinically establish that high mitochondrial content (85) is a functional biomarker of aggressive tumor progression and metastasis, as well as poor prognosis and reduced overall survival. In further support of these clinical observations, MT-CO2 is over-expressed by >20-fold in an hTERT-enriched sub-population of breast cancer stem cells (86). Pharmacologically, MT-CO2 is effectively targeted by the FDA-approved antibiotic Doxycycline (87), which behaves as an inhibitor of mitochondrial protein translation and prevents ATP production (87), ultimately blocking metastasis in preclinical models (54). Therefore, Doxycycline may also provide a therapeutic solution for inhibiting MT-CO2 in breast cancer patients, to help prevent disease progression.

Taken together, these multiple lines of experimental evidence are all consistent with the idea that mitochondrial ATP-depletion therapy should be pursued as a viable means to provide metastasis prophylaxis in cancer patients.

## Author Contributions

ML wrote the first draft of this review article, which was then further edited by MF, BÓ, and FS. MF prepared the figures, which were edited by BÓ, ML, and FS. All authors contributed to the article and approved the submitted version.

## Funding

This work was supported by research grant funding, provided by Lunella Biotech, Inc. (to FS and ML). The funder was not involved in the study design, collection, analysis, interpretation of data, the writing of this article or the decision to submit it for publication.

## Conflict of Interest

ML and FS hold a minority interest in Lunella Biotech, Inc.

The remaining authors declare that the research was conducted in the absence of any commercial or financial relationships that could be construed as a potential conflict of interest.

The reviewer, SF, declared a past co-authorship with one of the authors, ML, to the handling editor.

## Publisher’s Note

All claims expressed in this article are solely those of the authors and do not necessarily represent those of their affiliated organizations, or those of the publisher, the editors and the reviewers. Any product that may be evaluated in this article, or claim that may be made by its manufacturer, is not guaranteed or endorsed by the publisher.
